# No evidence of higher peak heat tolerance in the afternoon or with opportunity for thermal behaviour during low‐intensity work in uncompensable heat

**DOI:** 10.1113/EP092803

**Published:** 2026-09-15

**Authors:** Tiarna Stothers, Travis Dylan Gibbons, Kate Nicole Thomas, Emma Jones, Holly Ann Campbell, James David Cotter

**Affiliations:** ^1^ School of Physical Education, Sport and Exercise Sciences University of Otago Dunedin New Zealand; ^2^ Department of Surgical Sciences University of Otago Dunedin New Zealand; ^3^ Centre for Heart, Lung and Vascular Health Faculty of Health and Social Development, Okanagan Campus Kelowna British Columbia Canada

**Keywords:** hyperthermia, thermal behaviour, thermal discomfort, thermal tolerance

## Abstract

Heat tolerance is known to be limited by high brain temperature, but most findings pertain to strenuous exercise following artificially altered baseline temperature or with limited opportunity for behaviour. Therefore, we investigated the hypotheses that thermal perceptions, hyperventilation and cerebrovascular responses would be exacerbated in: (1) the afternoon relative to morning; and (2) the absence of opportunity for behavioural thermoregulation. Thirteen habitually active participants (seven females) cycled at 25% peak power wearing a water‐perfused suit (∼48°C) until volitional tolerance on three occasions, once in the morning (AM) and twice in the afternoon (PM), with or without access to face fanning with mist (∼1.7 m/s). Heating took ∼2 h. Core temperature (*T*_c_) was 0.35°C lower in AM at baseline (95% confidence interval: −0.55°C, −0.16°C; *P* < 0.001, Hedges’ *g* = 1.673) but not at tolerance (39.10°C ± 0.71°C vs. 39.11°C ± 0.50°C; *P* = 0.947, *g *= 0.024), hence the rise was greater (95% confidence interval: 0.00°C, 0.80°C; *P* = 0.047, *g *= 0.651). The fan did not confer higher *T*_c_ tolerance (95% confidence interval: −0.44°C, 0.24°C; *P* = 0.764, *g *= 0.050). The modelled *T*_c_ thresholds for heat discomfort and hyperventilation were ∼0.23°C lower in AM, but sensitivities did not differ reliably or with fan availability (*P* ≥ 0.098). Internal carotid artery flow was similar across time of day (*P* = 0.904) and showed little effect of heating. Changes in estimated intracranial pressure did not differ reliably between conditions. In conclusion, thermal reserve was smaller in PM by virtue of higher baseline, and psychophysical, ventilatory and cerebrovascular responses were not reliably different. For most individuals, an opportunity for behaviour did not improve discomfort, hyperventilation or tolerance.

## Abstract (highlights)

- **What is the central question of this study?**
  Is peak heat tolerance or its associated hyperventilation affected by either diurnally regulated differences in core temperature or research‐imposed restrictions on behavioural thermoregulation?
- **What is the main finding and its importance?**
  During light exercise with high skin temperature, peak tolerable core temperature was not shown to depend on diurnal thermoregulation, hence thermal reserve was lower in the afternoon. Thresholds for heat‐induced hyperventilation and discomfort approximated the resting core temperature in both the morning and the afternoon. An opportunity for behavioural thermoregulation (tested in the afternoon) did not consistently reduce thermal discomfort or hyperventilation or increase tolerable core temperature.

## 1 INTRODUCTION

Heat stress caused by physical exertion in hot or humid environments often causes a behavioural reduction in work rate well before maximal tolerable core temperature (*T*_c_) is reached (Periard et al., 2021; Schlader, Stannard et al., 2011; Tatterson et al., 2000). Nevertheless, research on animal models (Caputa et al., 1986; Walters et al., 2000) and humans (González‐Alonso et al., 1999) indicates that heat intolerance is precipitated by high *T*_c_ (González‐Alonso et al., 1999), particularly brain temperature (Caputa et al., 1986; Walters et al., 2000), although high skin temperature and wettedness, cardiovascular strain and dehydration have modulatory roles (Periard et al., 2021; Vargas et al., 2020).

Despite the known importance of *T*_c_, it is still unknown whether heat intolerance is limited by the rise in *T*_c_ or by the maximal *T*_c_ obtained. Artificially manipulating initial *T*_c_ by precooling and preheating (Booth et al., 1997; González‐Alonso et al., 1999) has demonstrated the importance of maximal *T*_c_, but this also uncouples *T*_c_ from its usual autonomic and behavioural thermoregulatory control. Studies that have examined natural differences in *T*_c_ (i.e., related to diurnal changes) show that morning *T*_c_ is significantly lower at the onset of exercise, but at exhaustion the final *T*_c_ reached is either not different (Hobson et al., 2009; Otani et al., 2020) or remains lower in accordance with starting temperature (Reilly & Garrett, 1998) when exercising at a high workload [i.e., 65%–70% of peak oxygen uptake (${\dot{V}}_{O_{2}\mathrm{peak}}$)]. Peak heat tolerance under high workloads is a valid and valuable context but is likely to involve more non‐thermal mediators of fatigue associated with inability to maintain the workload.

Heat stress incurs heat‐induced hyperventilation, reducing cerebral blood flow (CBF). Insufficient or excessive CBF could limit heat tolerance by causing cerebral hyperthermia, cerebral hypoxia or excessive intracranial pressure (ICP) (Bain et al., 2013; Gibbons et al., 2021; Nybo et al., 2002; Oshorov et al., 2021; Sharma, 2006). During hyperthermia, ICP increases owing to an increased permeability of the blood–brain barrier, increasing fluid volume and movement into cerebral tissue and causing cerebral oedema, ischaemia and, in severe cases, neural cell damage (Kiyatkin & Sharma, 2009, 2019; Kiyatkin et al., 2007; Lin & Lin, 1992; Sharma, 2006). The ICP response to heat stress remains under‐studied in healthy humans owing to difficulties in measurement, but the increase appears to occur at physiologically relevant levels of heat strain, i.e., *T*_c_ < 40°C. It has been proposed that hyperventilation might serve to protect heat‐induced intracranial hypertension by reducing intracranial volumes; however, evidence is indirect and inconclusive (Gibbons et al., 2021).

A confounding factor in most heat tolerance research might be the exclusion of opportunity for behavioural thermoregulation. Wet skin and elevations in skin temperature (*T*_sk_) and *T*_c_ can all drive thermal discomfort and worsen overall feeling state (Fukazawa & Havenith, 2009; Schlader et al., 2010; Vargas et al., 2020) and could limit peak heat tolerance during studies imposing heat stress passively (e.g., via a perfusion suit) or in conjunction with exercise. For example, heat discomfort can contribute to stress‐induced hyperventilation (Lucas et al., 2015), but the relationship between behaviour, thermal discomfort and hyperventilation during heat exposure is largely unstudied. The inclusion of behavioural cooling mechanisms (i.e., using a fan or cool mist) has significantly reduced thermal discomfort during exercise in temperate and hot environments (Mündel et al., 2007; Vargas et al., 2019). Although acute skin cooling can reduce the stimulus to hyperventilate (Lucas et al., 2010), it remains unclear whether this response is purely physiological (i.e., thermoafferent or possibly also via cardiovascular effects) or is psychophysical. The facial region has high alliesthesial thermosensitivity during heat stress (Cotter & Taylor, 2005; Ramanathan, 1964) and therefore stands to contribute disproportionately to thermal discomfort, without necessarily affecting *T*_c_ (Wang et al., 2023).

Thus, the purposes of this study were to determine: (1) whether the diurnal naturally occurring change in *T*_c_ affects peak heat tolerance (when metabolic homeostasis is less compromised); and (2) what role, if any, thermal discomfort has on physiological mechanisms involved in reaching maximal heat tolerance. We hypothesised that: (1) in conjunction with less *T*_c_ reserve in the afternoon, hyperventilation, thermal discomfort and reduced cerebral perfusion would occur earlier (i.e., with less rise in *T*_c_) and be more pronounced when compared with the morning; and (2) the opportunity for behavioural action would reduce psychophysical strain, hyperventilation and attenuation of CBF and would increase heat tolerance (indicated by higher peak *T*_c_) during incremental heating at a low work intensity. We further anticipated (based on the study by Gibbons et al., 2021) that ICP, measured indirectly, would increase to a common level independently of starting or ending *T*_c_, CBF or ventilatory responses.

## 2 MATERIALS AND METHODS

### 2.1 Ethical approval

Ethical approval was granted by the Institutional Human Ethics Committee at the University of Otago (H20/031) and conformed to the *Declaration of Helsinki*. This study was registered retrospectively in the Australian New Zealand Clinical Trials Registry (trial ID ACTRN12621001197820), because recruitment began prior to registration. Written informed consent was obtained from participants before testing started. Data will be made available on reasonable request.

### 2.2 Experimental design

This was a cross‐over design, with participants completing three experimental conditions, each consisting of semi‐recumbent cycling at a constant work rate (25% peak work rate) in a hot‐water‐perfused suit until volitional thermal tolerance (or *T*_c_ of 40°C). Conditions differed only in whether they started in the morning (AM) or afternoon (PM) and whether the afternoon session included the opportunity for behavioural thermoregulation, with a fan and cool mist directed onto the face (PM+Fan). Conditions were randomised and separated by a minimum of 5 days to minimise fatigue or adaptation. Participants were blinded to the hypotheses prior to the study, in addition to their *T*_c_ and the timing of measurements during the experimental conditions. Prior to the first experimental condition, participants underwent a maximal work rate test on a semi‐recumbent cycle ergometer (Velotron, Racermate, Seattle, WA, USA) in a temperate environment, followed by familiarisation with the experimental protocol. Data collection occurred in winter–early spring (June–October), when mean monthly air temperatures ranged from 6.7°C to 10.4°C. Participants were advised to avoid other forms of heat exposure for the duration of their participation and were asked upon arrival on the testing day whether they had adhered to this.

### 2.3 Participants

Thirteen aerobically fit volunteers were participants; six males and seven females (age 26 ± 5 years, height 172 ± 8 cm, mass 73.6 ± 11.2 kg, peak power output 256 ± 45 W and predicted ${\dot{V}}_{O_{2}\mathrm{peak}}$ 54 ± 11 mL/min/kg). Participants were physically active (>3 times per week for 30 min or more) and had no contraindications to moderate heat stress or exercise. Females were either naturally menstruating (*n* = 5) or using oral contraception (*n* = 1, combined monophasic pill with synthesised oestrogen and progestogen) or an implant (*n* = 1, Jadelle, levonorgestrel‐releasing implant) and were tested during their luteal phase (confirmed via ovulation test) or active pill phase. Participants were asked to replicate food and caffeine intake (quantity and time) for the 12 h prior to testing (previous two meals). To standardise hydration status, participants were asked to avoid alcohol consumption for 24 h prior to testing and to consume 10 mL/kg of water on the night before and the morning of testing. Participants were also advised to refrain from doing any abnormally strenuous exercise for 24 h prior to the test and to avoid exposure to hot environments during the study.

### 2.4 Experimental protocol

#### 2.4.1 Familiarisation

Participants underwent a familiarisation session after their incremental‐to‐peak exercise test. This involved exposure to the heat stress (described below), including 25% of peak work rate and collection of the cerebrovascular, cardiorespiratory and perceptual variables described below.

#### 2.4.2 Exercise protocols

**FIGURE 1:**
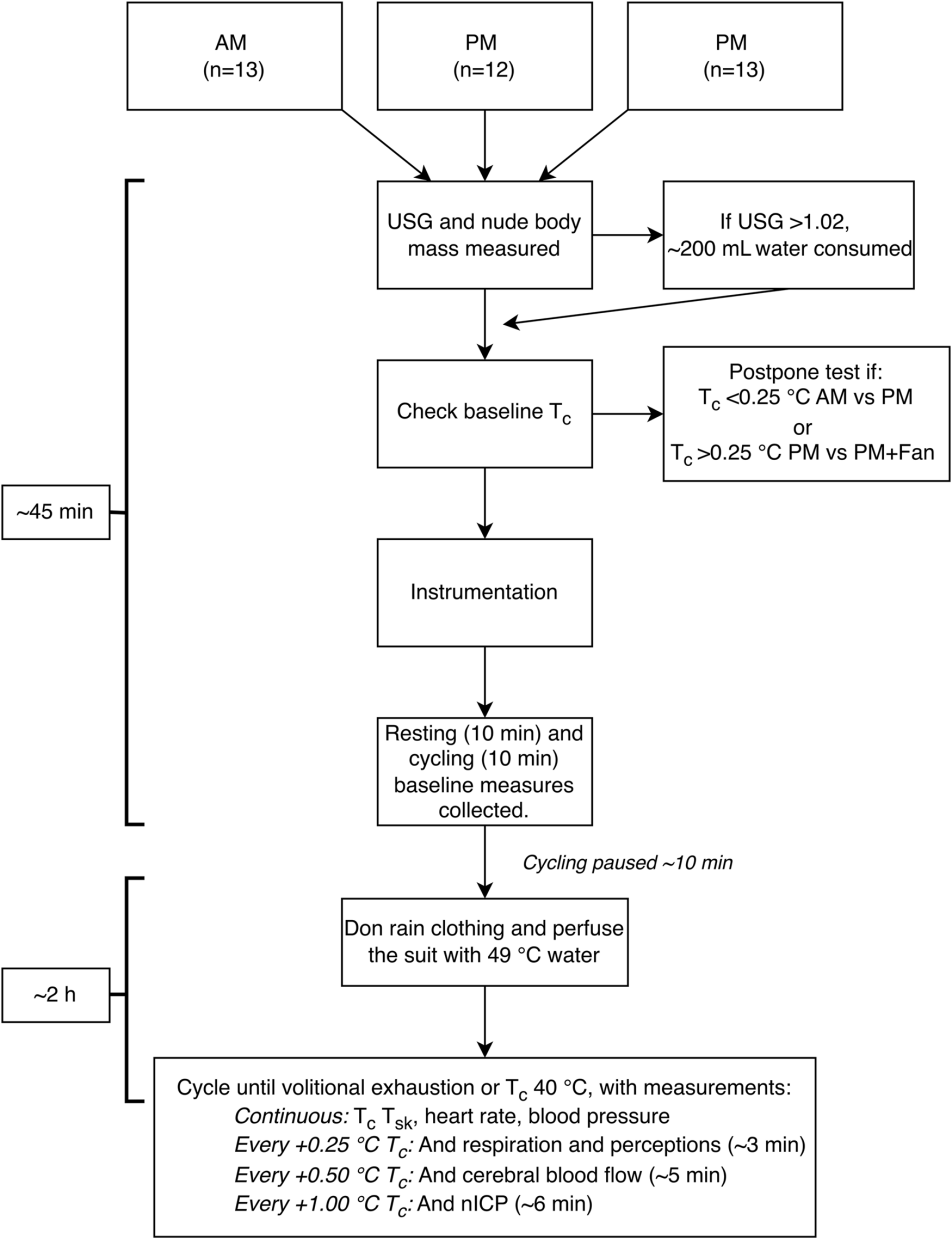
Flow chart demonstrating the time line of the experimental conditions. Abbreviations: AM, morning or lower baseline condition; nICP, non invasive estimation of Intracranial Pressure; PM, afternoon or higher baseline condition; PM+Fan, afternoon condition with fan; *T*_c_, core temperature; *T*_sk_, skin temperature; USG, urine specific gravity.

The protocol is illustrated in Figure 1. On arrival at the laboratory, participants gave a urine sample to measure urine specific gravity, inserted a rectal thermistor and recorded nude body mass. If urine specific gravity was >1.020, a minimum of 200 mL of water was provided for ingestion, although more was provided if requested (the water mass ingested was 368 ± 209 mL). At this point, *T*_c_ was noted to determine whether there was a different (>0.25°C; AM vs. PM) or similar (<0.25°C; PM vs. PM+Fan) temperature to proceed with the appropriate condition. The condition was rescheduled for the following day if *T*_c_ seemed atypical (not demonstrating appropriate difference or similarity). If *T*_c_ was appropriate, participants were fitted with cardiorespiratory and thermometry equipment and donned the water‐perfused suit. Resting baseline measures were collected at this stage, followed by exercise baseline (ExBL) measures. ExBL consisted of starting semi‐recumbent cycling at 25% of peak work rate, to quantify the change in cardiorespiratory, cerebrovascular and perceptual changes with exercise. No water flowed through the suit for the collection of pre‐exercise and ExBL measures, hence *T*_c_ remained stable or drifted naturally. Following baseline and ExBL measures, participants donned a waterproof coat and trousers over the water‐perfused suit, which was then perfused with water at 48°C–49°C, creating uncompensable heat stress. Participants cycled on a semi‐recumbent cycle ergometer at 25% of their individual peak work rate (57 ± 15 W) until either volitional thermal tolerance or *T*_c_ reached 40°C. Participants were encouraged to drink 200 mL of water following the collection of the perceptual measures (+0.25°C increments in *T*_c_; to match approximated sweat loss), but additional water was also given upon request. The mass and timing of water consumption were recorded and replicated across conditions to minimise dehydration to <2% loss of body mass. The temperature of drinking water was always kept within 4°C (usually 1°C) of *T*_c_ to remove the effect that cool water could have on thermal perception.

The afternoon condition with the fan (PM+Fan) allowed participants to use a button‐automated fan and mist directed at the face, which, when pressed, was activated for 30 s. The fan was positioned ∼1 m in front of, and level with, the participant's face. The fan speed was fixed, which delivered an effective air velocity of ∼1.7 m/s. Prior to beginning data collection in each PM+Fan condition, participants received a standardised set of instructions on how to control the fan to make them feel more comfortable and were given a brief trial period to get used to the sensation before the onset of heating. The misting spray was temporarily deactivated during the collection of cerebrovascular and respiratory measures for reasons of electrical safety and protection, but this did not affect the ability of participants to use the fan throughout.

### 2.5 Measurements

#### 2.5.1 Thermometry

The *T*_c_ was measured in the rectum, 10–15 cm past the anal sphincter, using thermistors (Mon‐A‐Therm, Covidien, Mansfield, MA, USA). The rectal index was considered appropriate for reasons explained in Discussion. Skin temperature was measured at four standardised sites (thigh, forearm, scapula and forehead) using insulated surface thermistors (Skin Thermistor EUS‐U‐V5‐V1; Grant Instruments, Cambridge, UK) taped on the skin. Temperatures were recorded on a portable logger (Squirrel v.2010, Grant Instruments) at 10 s intervals and were visible to researchers at all times.

#### 2.5.2 CBF and optic nerve sheath diameter

Duplex ultrasound (Terason uSmart t3300, Teratech) was used to measure the diameter and blood velocity within the right internal carotid artery (ICA) and vertebral artery (VA). The ICA was insonated >1 cm above the bifurcation of the common carotid artery to reduce noise from turbulent blood flow (Thomas et al., 2015) and was measured during baseline exercise, at each +0.5°C increase in *T*_c_ and at tolerance or termination of the test. Optic nerve sheath diameter (ONSD) provided a non‐invasive measurement of intracranial pressure (nICP). ONSD was insonated using ultrasound in both eyes (over closed eyelids) using sagittal and transverse planes during baseline exercise, at each +1°C increase in *T*_c_ and at tolerance or test termination. All ultrasound measures were performed by one of two experienced researchers to aid reliability.

#### 2.5.3 Perceptions

Thermal sensation (1–13; extremely cold to extremely hot), thermal discomfort (1–9; comfortable to extremely uncomfortable; extended from Gagge et al., 1969), overall feeling (−5 to +5; very bad to very good) and rating of perceived exertion (RPE; 6–20, no exertion to maximal exertion; Borg, 1982) were measured upon arrival, during baseline exercise, at each +0.25°C increase in *T*_c_ and at volitional tolerance or test termination. Additionally, participants were asked to explain the reason behind volitional termination of the test (e.g., was it the exercise, the heat, the breathing or something else?).

#### 2.5.4 Quantifying behavioural thermoregulation

Behavioural thermoregulation was indicated by the number of times the participant pressed the push button switch (MLA92/D, ADInstruments Dunedin New Zealand) connected via DIN input to PowerLab (PowerLab/16SP, ADInstruments). When pressed, the button provided a 1 V output (recorded in LabChart software; LabChart v.8, ADInstruments) that switched on a misting fan for 30 s. During this 30 s ‘on’ period of the fan, button presses were not accumulative (i.e., the button could not restart the fan within the 30 s), but these futile presses were still recorded. The number of button presses was recorded continuously in LabChart software (LabChart v.8, ADInstruments) and interpreted as number of presses per minute, which served as a quantitative measure of behaviour.

#### 2.5.5 Cardiorespiratory

Cardiorespiratory measures were recorded using LabChart software (LabChart v.8, ADInstruments). Heart rate and blood pressure were measured continuously using an ECG (ADInstruments) and finger cuff photoplethysmography (Finometer, Finapres Medical Systems, Amsterdam, The Netherlands). The finger cuff measurement was calibrated against three manual brachial blood pressure measurements. Respiratory measures [i.e., ventilation (BTPS), breathing frequency, tidal volume and pressure of end‐tidal CO_2_ ($P_{\mathrm{ET},CO_{2}}$)] were monitored and recorded using a flowmeter (Turbine 2000, Cosmed, Rome, Italy), Nafion quick dry sample line and gas analyser (PowerLab/16SP, ADInstruments). At the start of every condition, the flowmeter and gas analyser were calibrated to a known volume (3.00 L) and gas concentration (16% O_2_ and 5% CO_2_). Gas concentrations were checked periodically for drift owing to saturation of Nafion with prolonged sampling. Respiratory variables were collected at rest (∼5–10 min), during baseline exercise (∼10 min), at each +0.25°C increase in *T*_c_ and at volitional tolerance (for ≥3 min).

### 2.6 Data analysis

#### 2.6.1 Calculations

The ONSD recordings were analysed to produce six images (from each video recording, thus six diameters) that best showed both walls of the nerve sheath. The diameter was measured 3 mm behind the retina of the eye and processed using ImageJ (NIH, Bethesda, MD, USA). The six diameters were averaged to produce one diameter to represent each heating stage (i.e., ExBL, +1°C, +2°C, tolerance). The ONSD was then converted to a pressure (nICP) using the following formula from Robba et al. (2017):

$$\mathrm{nICP}\;=\left(\mathrm{ONSD}\;\times \;5\right)\;-\;13.92.$$

The diameter and blood velocity of the ICA and VA were measured using edge‐tracking software (Cardiovascular Suite v.3.5.3, Quipu, Pisa, Italy), from which blood flow for each vessel was calculated using the following formula:

$$\begin{array}{ccc} & & \left(\mathrm{peak}\;\mathrm{envelope}\;\mathrm{blood}\;\mathrm{velocity}/2\right)\times \left[\pi {\left(0.5\times \mathrm{diameter}\right)}^{2}\right]\\ & & \times \;60\left(\times \;2\;\mathrm{for}\;\mathrm{bilaterality}\right) \end{array}$$

Mean skin temperature was adapted from Ramanathan (1964) using the following formula:

$$\left(\mathrm{forearm}\;\times \;0.19\right)+\left(\mathrm{head}\;\times \;0.07\right)+\left(\mathrm{thigh}\;\times \;0.39\right)+\left(\mathrm{scapula}\;\times \;0.35\right).$$

Slopes, intercepts and thresholds were calculated for each session using Microsoft Excel (Microsoft, WA, USA) with the following formulae:

$$\mathrm{Slope}=\frac{\sum \left(x-\bar{x\;}\right)\left(y-\bar{y}\right)}{{\sum \left(x-\bar{x\;}\right)}^{2}}$$

$$\mathrm{Intercept}=\bar{y}-\left(\mathrm{slope}\times \bar{x\;}\right)$$

$$T_{c}\;\mathrm{thresholds}=\frac{\mathrm{baseline}-\mathrm{intercept}}{\mathrm{slope}}$$

#### 2.6.2 Statistics

A sample size calculation was performed based on data from a similar study (Gibbons et al., 2021). To detect a mean increase in ventilation of 3.0 L/min between conditions, assuming an SD of the within‐subject change of 3.3 L/min, 12 participants were required to provide 80% power with a significance level of 0.05. To account for drop‐out and incomplete data, 13 participants were recruited for this study. Data were collated using Microsoft Excel (Microsoft, WA, USA). Inferential analyses were undertaken for each of the two research questions, i.e., (1) effect of different baseline *T*_c_ (>0.25°C for AM vs. PM comparison); and (2) effect of behavioural opportunity (PM vs. PM+Fan). For each question, a linear mixed‐model ANOVA was used to test main and interaction effects of condition and time, using R (R Development Core Team, 2020). Fitness (estimated, relative ${\dot{V}}_{O_{2}\mathrm{peak}}$), order of session, and sex were included as covariates and not centred. Tukey's method (emmeans) was used for *post hoc* testing and adjustment of confidence intervals for two estimates. Ventilatory (ventilation and $P_{\mathrm{ET},CO_{2}}$) and perceptual responses were modelled as a function of the *T*_c_ and the change in *T*_c_ to determine the response characteristics (i.e., thresholds, slopes and peak tolerance) and compared using paired *t*‐tests. The thresholds were modelled only if *r* was >0.79. Results obtained were considered significant when *P* < 0.05. Figures were produced using GraphPad Prism (v.9.3.1, GraphPad Software, La Jolla, CA, USA). All analysed data are presented as the mean ± SD, with 95% confidence intervals (CI) and within‐subjects Hedges’ *g* effect sizes (*g*) provided for comparisons. For efficiency of space and the number of figures and tables, data for all three conditions are shown within the same figures and tables in the Results section, but analyses were limited to the two comparisons described above, hence AM is never compared against PM+Fan in tables or figures.

## 3 RESULTS

### 3.1 Participant characteristics and standardisation

Participants reported complying with standardisation procedures. Food diaries were collected to ensure that adequate replication took place. Urine specific gravity indicated that most participants were euhydrated prior to each testing condition (AM, 1.017 ± 0.009; PM, 1.011 ± 0.009; PM+Fan, 1.012 ± 0.009). Six participants in AM, three in PM and four in PM+Fan had urine specific gravity > 1.020 and were given 368 ± 209 mL of water prior to commencing of heating. Body mass decreased by 1.8% ± 0.9% and was not different across the time of day (AM, −1.8% ± 0.8%; PM, −1.7% ± 0.9%; *P* = 0.910, *g *= 0.150) or the behaviour conditions (PM+Fan, −1.8% ± 1.0%; *P* = 0.578, *g *= 0.244). Flow rate of water through the suit was 1.4 ± 0.33 L/min, and water temperature remained constant between 48°C and 49°C.

Twelve of the 13 participants completed all three trials. The participant who completed only two conditions (AM and PM+Fan) had a matched baseline *T*_c_ and was therefore included in the behavioural analysis only. Two trials were postponed owing to baseline temperatures deemed as atypical (i.e., afternoon *T*_c_ < 36.5°C). One participant had a cooler baseline *T*_c_ in the afternoon than in the morning but had complied with standardisation requirements, hence both trials were included, with their cooler trial designated as ‘AM’ (i.e., the lower regulated baseline *T*_c_).

### 3.2 Thermometry and thermal tolerance

**FIGURE 2:**
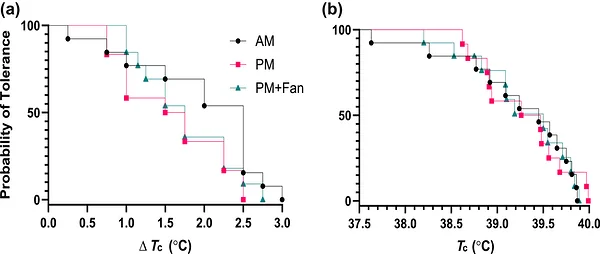
Heat tolerance shown as survival curves as a function of change in *T*_c_ (Δ*T*_c_; a) or *T*_c_ (b). *n* = 12 for AM, *n* = 13 for PM and PM+Fan. Abbreviations: AM, morning or lower baseline condition; PM, afternoon or higher baseline condition; PM+Fan, afternoon condition with fan; *T*_c_, core temperature.

Overall, 82% of exposures were stopped owing to participants declaring their limit of tolerance, and the other 18% were stopped within 0.2°C of the 40°C *T*_c_ ethical cut‐off (for tolerance plots, see Figure 2a,b). In only one of these end‐point‐terminated trials did the participant not believe that they were at or near thermal tolerance (i.e., at least thermal discomfort of ‘very uncomfortable’ and RPE ‘hard’). The average duration of heating, between recording of exercise baseline and final (tolerance) data, was ∼2 h, with AM being an average of 17 ± 42 min longer than PM (Table 1; *g *= 0.373) and PM being an average of 18 ± 24 min shorter than PM+Fan (*g *= 0.707).

**TABLE 1:** Thermometry and cardiorespiratory variables at exercise baseline and heat tolerance.

	AM		PM		PM+Fan		AM vs. PM (*P*‐value)		PM vs. PM+Fan (*P*‐value)	
Variable	ExBL	Tolerance	ExBL	Tolerance	ExBL	Tolerance	ExBL	Tolerance	ExBL	Tolerance
*T*_c_, °C	36.90 ± 0.32	39.15 ± 0.68	37.26 ± 0.26	39.09 ± 0.51	37.24 ± 0.27	39.20 ± 0.55	<0.001	0.947	0.962	0.764
Δ*T*_c_/Δ*t*, °C/h	1.18 ± 0.47		1.04 ± 0.31		0.98 ± 0.31		0.350		0.288	
Duration, min	123 ± 47		107 ± 31		125 ± 41		0.194		0.019	
*T*_sk_, °C	31.86 ± 0.90	38.94 ± 1.42	33.12 ± 0.83	39.02 ± 0.63	33.14 ± 0.85	38.97 ± 0.98	<0.001	0.238	0.382	0.201
Heart rate, beats/min	98 ± 14	163 ± 14	100 ± 9	166 ± 12	101 ± 9	159 ± 16	0.876	0.867	0.890	0.045
$\dot{V}$_E_, L/min	30 ± 5	47 ± 9	31 ± 5	55 ± 16	30 ± 4	47 ± 9	0.148	0.027	0.133	0.046
Tidal volume, L	1.4 ± 0.3	1.6 ± 0.4	1.4 ± 0.2	1.7 ± 0.5	1.4 ± 0.3	1.4 ± 0.4	0.619	0.256	0.806	0.030
Breathing rate, breaths/min	22 ± 4	33 ± 14	22 ± 4	35 ± 11	22 ± 4	35 ± 11	0.727	0.308	0.907	0.723
$P_{\mathrm{ET},CO_{2}}$, mm Hg	40.2 ± 3.6	31.7 ± 4.9	40.6 ± 3.0	30.3 ± 5.0	40.8 ± 2.6	33.7 ± 3.3	0.961	0.155	0.194	0.021
MAP, mm Hg	91 ± 11	88 ± 13	89 ± 7	85 ± 8	91 ± 8	92 ± 11	0.447	0.363	0.421	0.109

Abbreviations: AM, morning or lower baseline condition; ExBL, exercise baseline; MAP, mean arterial pressure; $P_{\mathrm{ET},CO_{2}}$, end‐tidal carbon dioxide pressure; PM, afternoon or higher baseline condition; PM+Fan, afternoon condition with fan; *T*_c_, core temperature; Tolerance, volitional tolerance or 40°C *T*_c_; T_sk_, mean skin temperature; $\dot{V}$_E_, ventilation.

**FIGURE 3:**
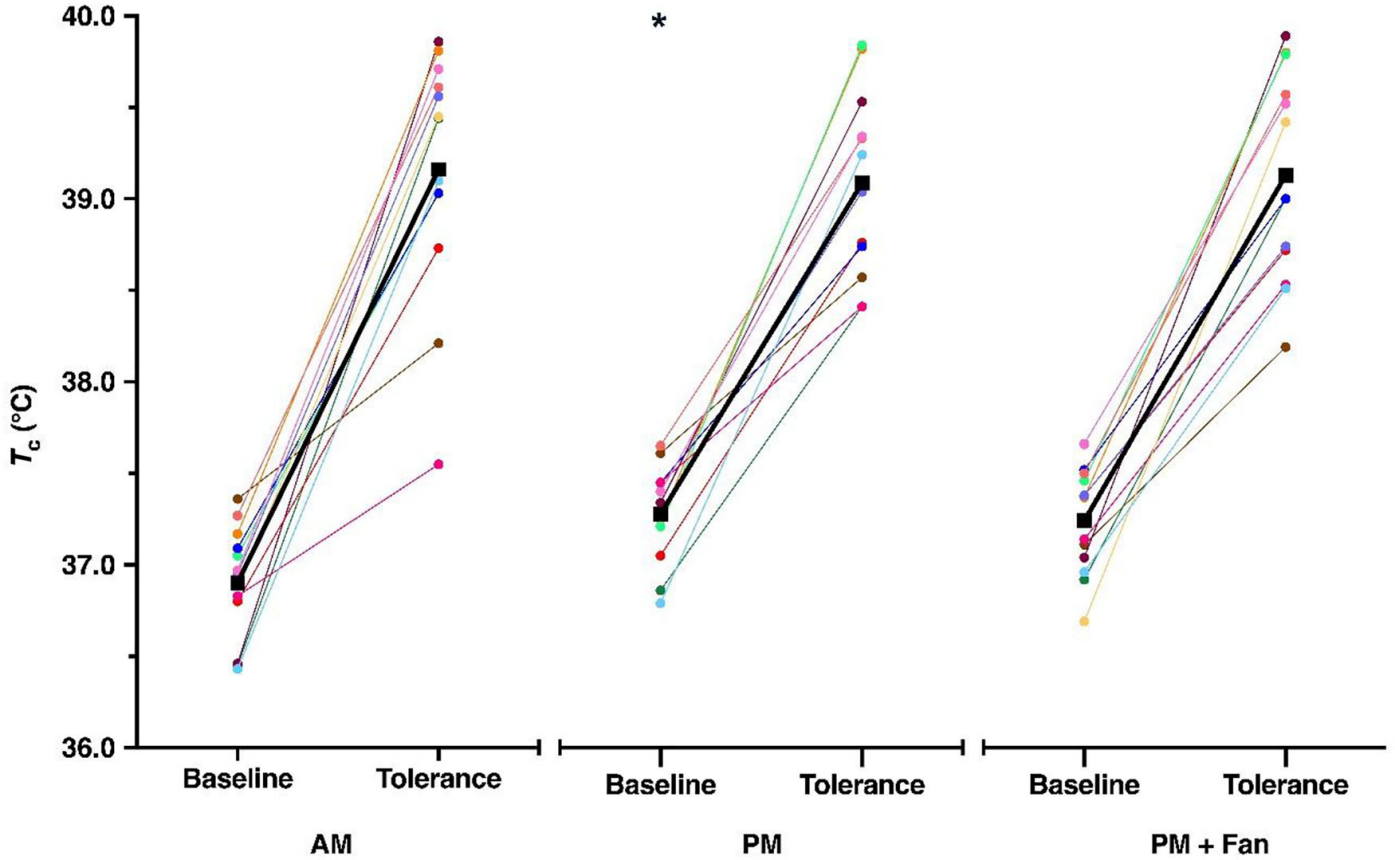
Core temperature (*T*_c_) at baseline and peak tolerance as a function of time of day (AM and PM; *n* = 12) or opportunity for behavioural thermoregulation (PM+Fan; *n* = 13). Each colour represents a participant or the mean response (black), which remains consistent across figures. *Significant difference from AM baseline; *P* < 0.05.

Baseline *T*_c_ was on average 0.35°C lower in AM compared with PM (95% Cl: −0.55, −0.16; *P* < 0.001, *g *= 1.673) but was not reliably different (0.01°C) between PM and PM+Fan (95% CI: −0.17, 0.22, *P* = 0.962, *g *= 0.024; Table 1). Maximum values of *T*_c_ reached were not different between AM and PM (95% CI: 0.55, −0.16; *P* = 0.947, *g *= 0.090) or between PM and PM+Fan (95% CI: −0.44, 0.24; *P* = 0.764, *g *= 0.050; Figure 3). Therefore, the rise in *T*_c_ was significantly greater in AM compared with PM (95% CI: 0.004, 0.80; *P* = 0.047, *g *= 0.651) but was not different between PM and PM+Fan (*g *= 0.021). The average difference in peak tolerable *T*_c_ of individuals between AM and PM was 0.41°C ± 0.33°C, which was smaller than the typical variability in tolerance between people (SD of 0.69°C in AM and 0.51°C in PM; Figure 3). Likewise, thermal reserve (Δ*T*_c_) for the two most tolerant participants was four times larger than for the two least tolerant participants in AM (Figures 2a and 3).

Baseline *T*_sk_ (Table 1) was 1.26°C lower in AM than PM (*P* < 0.001, *g *= 1.779) but was not different between PM and PM+Fan (0.01°C; *P* = 0.382, *g *= 0.072). End *T*_sk_ was not different between AM and PM (*P* = 0.238, *g *= 0.195) or between PM and PM+Fan (*P* = 0.201, *g *= 0.037; Table 1). The average forehead temperature across PM+Fan (31.81°C ± 1.23°C) was lower than in PM (35.12°C ± 0.65°C) by an average of 3.31°C ± 1.48°C (*P* < 0.001, *g = *2.104).

### 3.3 Cardiorespiratory responses

A summary of starting (ExBL) and tolerance data for cardiorespiratory measures is displayed in Table 1. The ventilation at tolerance was on average 17% higher in PM than AM (*P* = 0.027, *g *= 0.591) and 17% higher than in PM+Fan (*P* = 0.046, *g *= 0.508); the latter was attributable to higher tidal volume (by 21% on average) and resulted in more severe hypocapnia (by an average of 3.4 mm Hg).

**FIGURE 4:**
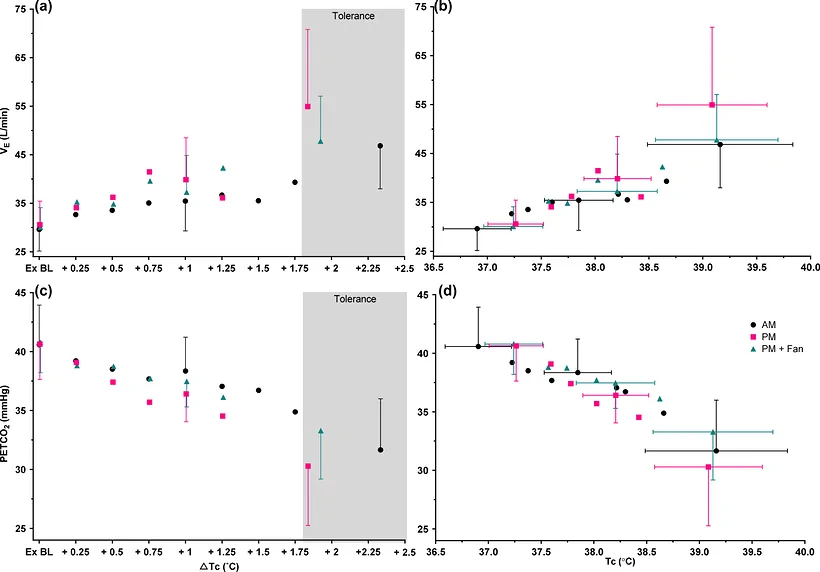
Summary of the ventilation ($\dot{V}$_E_; a, b) and end‐tidal carbon dioxide pressure ($P_{\mathrm{ET},CO_{2}}$; c, d) responses as a function of change in core temperature (Δ *T*_c_; a, c) or core temperature (*T*_c_; b, d). Data are the mean and SD; *n* = 12 for AM and *n* = 13 for PM and PM+Fan at Exercise Baseline and Tolerance, and *n* ≥ 10 during heat stress.

**FIGURE 5:**
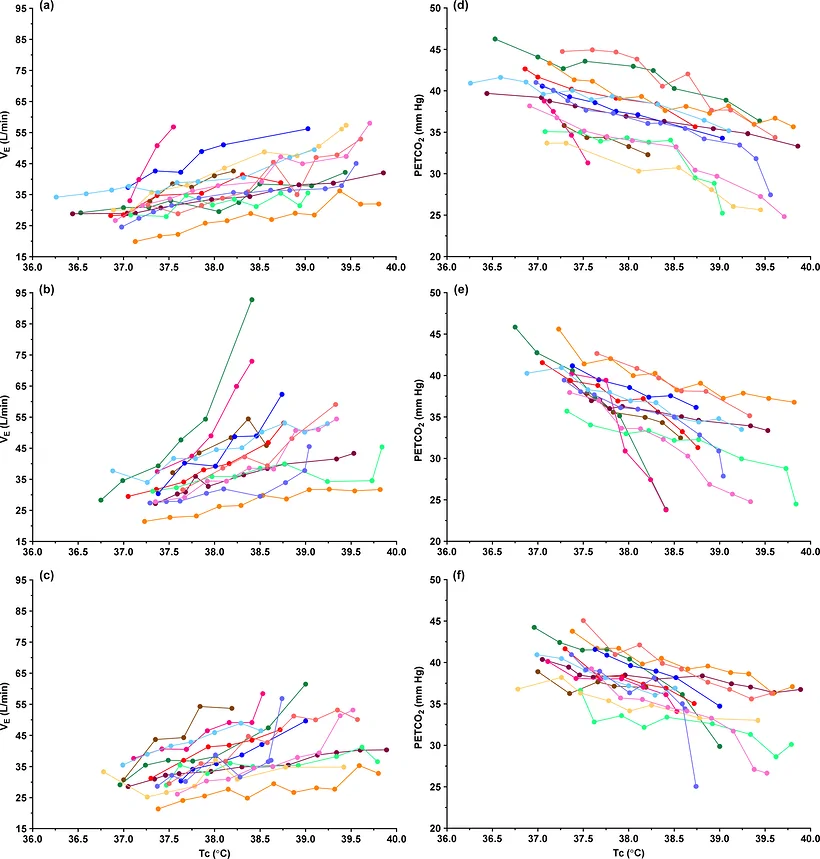
Individual ventilation ($\dot{V}$_E_) and end‐tidal carbon dioxide pressure ($P_{\mathrm{ET},CO_{2}}$) as a function of rising core temperature in the morning (AM; a, d), afternoon (PM; b, e) and afternoon with fan (PM+Fan; c, f).

**FIGURE 6:**
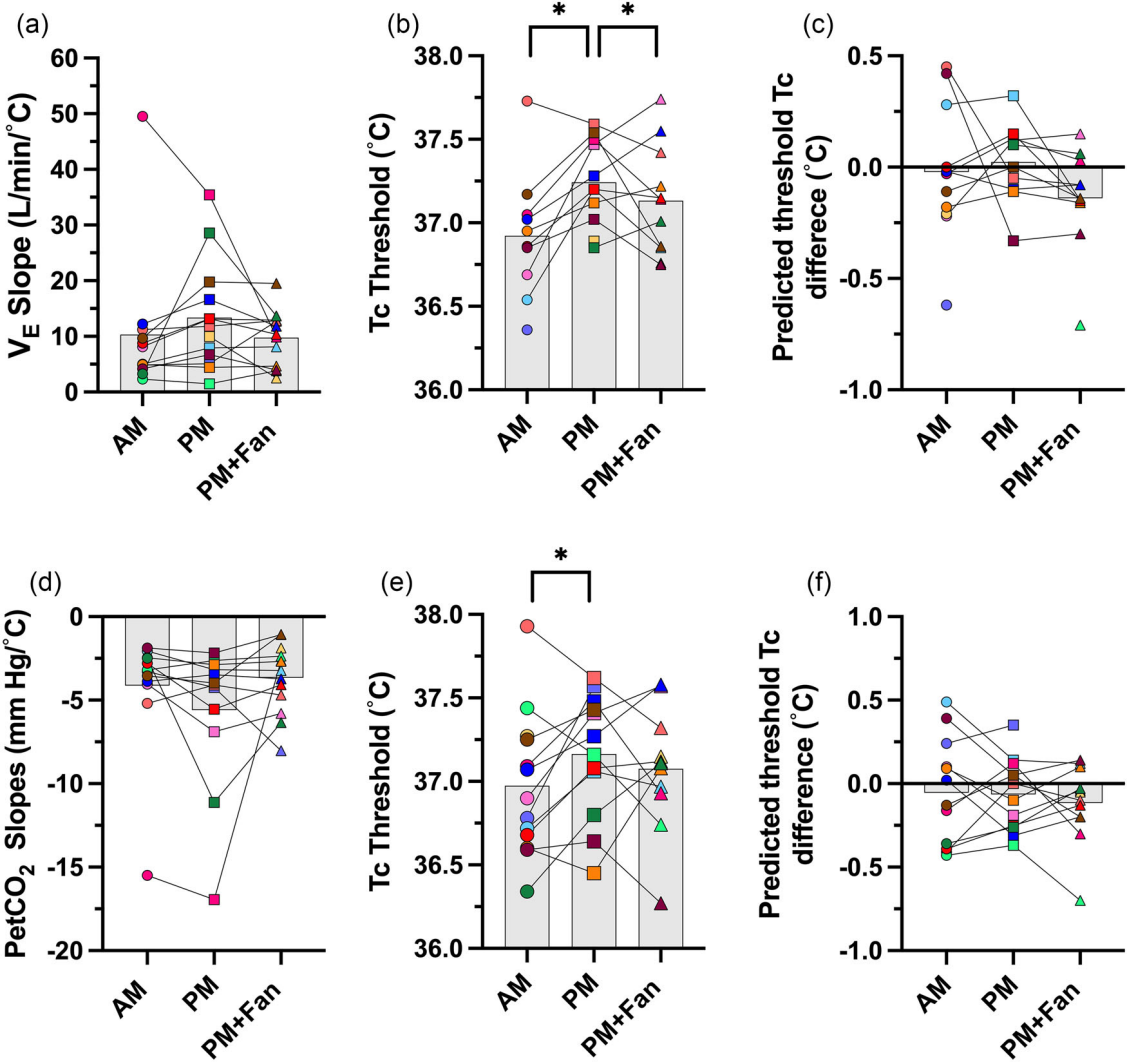
The ventilation ($\dot{V}$_E_) and corresponding end‐tidal carbon dioxide pressure ($P_{\mathrm{ET},CO_{2}}$) slopes (a), predicted core temperature thresholds (b) and offset of predicted threshold from baseline *T*_c_ (c) in morning (AM), afternoon (PM) and afternoon with fan (PM+Fan) conditions. *P < 0.05. Abbreviations: *T*_c_, core temperature.

Ventilation increased linearly, on average, with increasing *T*_c_ (Figure 4a,b), but a non‐linear increase reflecting clear hyperventilation preceding peak heat tolerance was evident in a minority of participants (Figure 5). The slope of the ventilation response relative to *T*_c_ (Figure 6a) was not significantly different between AM and PM (10.2 ± 12.7 vs. 12.4 ± 9.8 L/min/°C; *P* = 0.207, *g *= 0.648) or between PM and PM+Fan (9.8 ± 5.0 L/min/°C; *P* = 0.148, *g *= 0.446). The modelled *T*_c_ threshold for this increase in ventilation was significantly lower in AM than PM (36.92°C ± 0.38°C vs. 37.24°C ± 0.26°C; *P* = 0.037, *g *= 0.254) and in PM+Fan (37.1°C ± 0.3°C; *P* = 0.050, *g *= 0.326; Figure 6c).

The $P_{\mathrm{ET},CO_{2}}$ decreased with increasing *T*_c_ throughout all conditions, by −8.9 ± 1.3 mm Hg at tolerance (Figure 4c,d and Table 1) and, typically, in a linear manner (Figure 5). The slope or sensitivity of the $P_{\mathrm{ET},CO_{2}}$ response (Figure 6a) was not statistically different between AM and PM (−4.2 ± 3.7 vs. −5.5 ± 4.2 mm Hg/°C; *P* = 0.086, *g *= 0.512) or with facial cooling (PM+Fan: −3.7 ± 2.1 mm Hg/°C; *p* = 0.133, *g *= 0.472). Predicted thresholds were significantly lower in AM than PM (36.95°C ± 0.44°C vs. 37.16°C ± 0.35°C; *P* = 0.047, *g *= 0.416) but not lower with facial cooling (37.18°C ± 0.40°C; *P* = 0.695, *g *= 0.182; Figure 6b). The predicted thresholds for the onset of hyperventilation (i.e., increased ventilation and decreased $P_{\mathrm{ET},CO_{2}}$) approximated the baseline *T*_c_ in all conditions, on average (Figure 6c).

### 3.4 Cerebrovascular responses

A summary of the cerebrovascular variables is displayed in Table 2. The change in ICA flow across heating was 2% ± 17% in AM and −8% ± 17% in PM and did not differ reliably between AM and PM (95% Cl: −89, 62 mL/min; *P* = 0.904, *g* = 0.179). The apparently 5% greater reduction in ICA flow in PM+Fan than in PM (95% CI: 0.4, 149; *P* = 0.049, *g* = 0.198) was related to a lower ICA diameter at baseline (*P* = 0.033) and might therefore reflect sampling error. The nICP did not increase from ExBL to tolerance and did not differ between conditions (Table 2).

**TABLE 2:** Cerebrovascular variables at exercise baseline (ExBL) and limit of Tolerance for morning (AM), afternoon (PM) and afternoon with fan (PM+Fan).

	AM		PM		PM+Fan		AM vs. PM (*P*‐value)		PM vs. PM+Fan (*P*‐value)	
Variable	ExBL	Tolerance	ExBL	Tolerance	ExBL	Tolerance	ExBL	Tolerance	ExBL	Tolerance
ICA flow, mL/min	219 ± 46	209 ± 41	237 ± 64	211 ± 54	239 ± 62	223 ± 43	0.665	0.866	0.021	0.284
VA flow, mL/min	122 ± 42	131 ± 52	118 ± 39	128 ± 62	–	–	0.201	0.232	NA	NA
nICP, mm Hg	16.3 ± 2.7	16.4 ± 2.2	16.5 ± 2.7	16.6 ± 2.2	16.9 ± 2.5	17.8 ± 1.6	0.733	0.747	0.534	0.050

*Note*: Data are the mean ± SD for *n *= 11, except for *n *= 9 for VA and for ICA at Tolerance in AM.

Abbreviations: *d*, diameter; ExBL, exercise baseline; ICA, internal carotid artery; nICP, estimated intracranial pressure; VA, vertebral artery.

#### 3.4.1 Psychophysical

**FIGURE 7:**
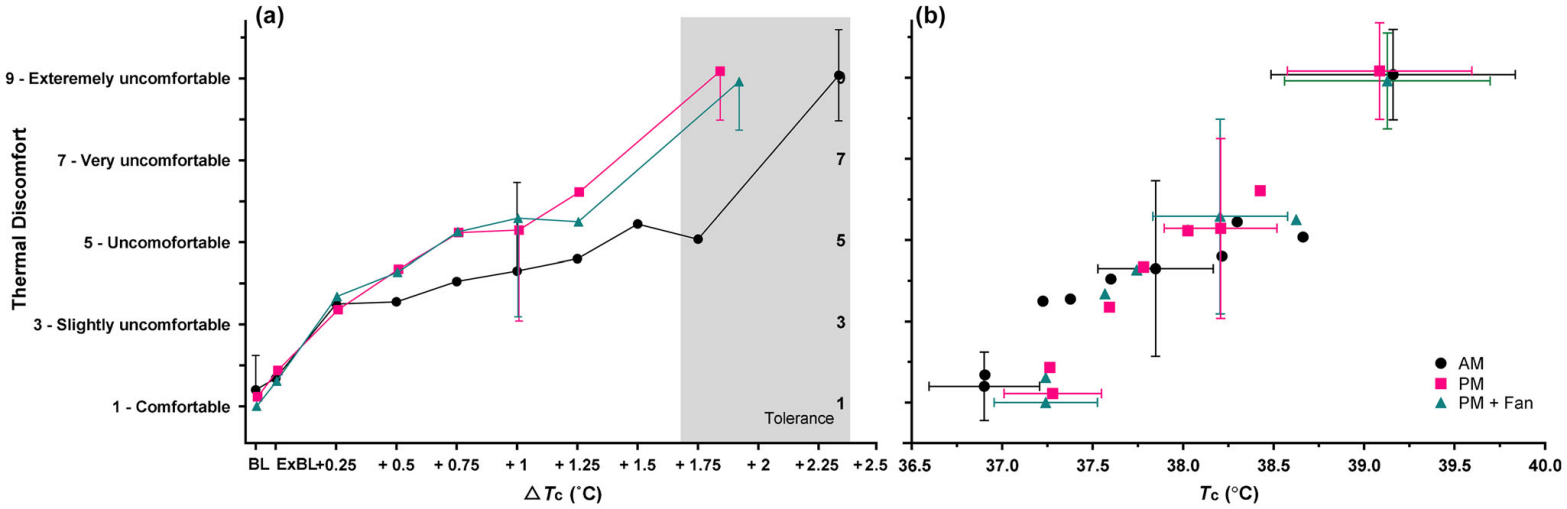
Thermal discomfort (1–10; comfortable to extremely uncomfortable) as a function of the change in core temperature (∆*T*_c_; a) or the core temperature (*T*_c_; b) from exercise baseline (ExBL). Data are the mean, with SD shown intermittently for visual clarity, for *n* = 12 for AM, *n* = 13 for PM and PM+Fan. The data are plotted in each condition while *n* ≥ 10, and tolerance includes all 12–13 participants. Abbreviations: *T*_c_, core temperature.

**FIGURE 8:**
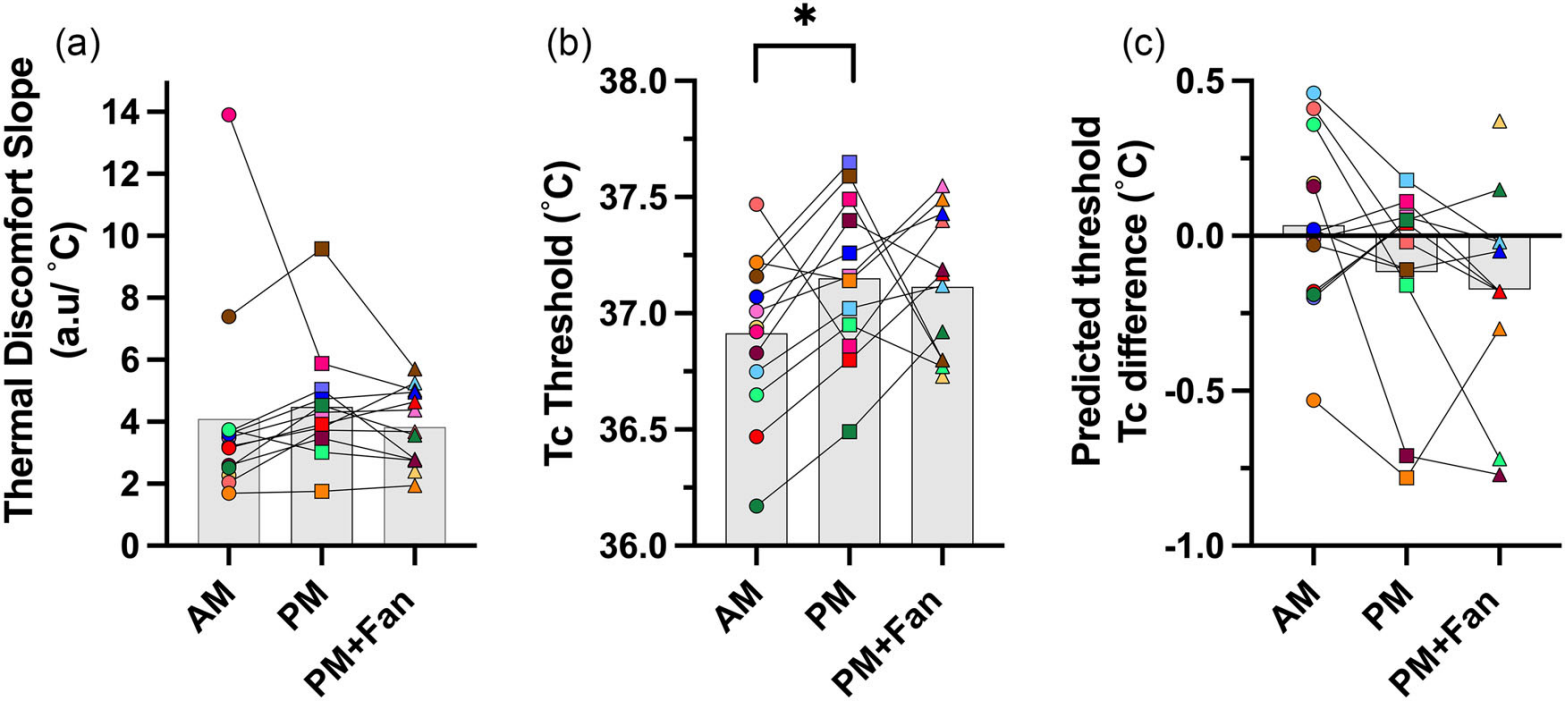
Thermal discomfort sensitivity (a), predicted thresholds (b) and offset of predicted *T*_c_ threshold from baseline *T*_c_ (c), for morning (AM), afternoon (PM) and afternoon with fan (PM+Fan). *P < 0.05. Abbreviations: *T*_c_, core temperature.

**FIGURE 9:**
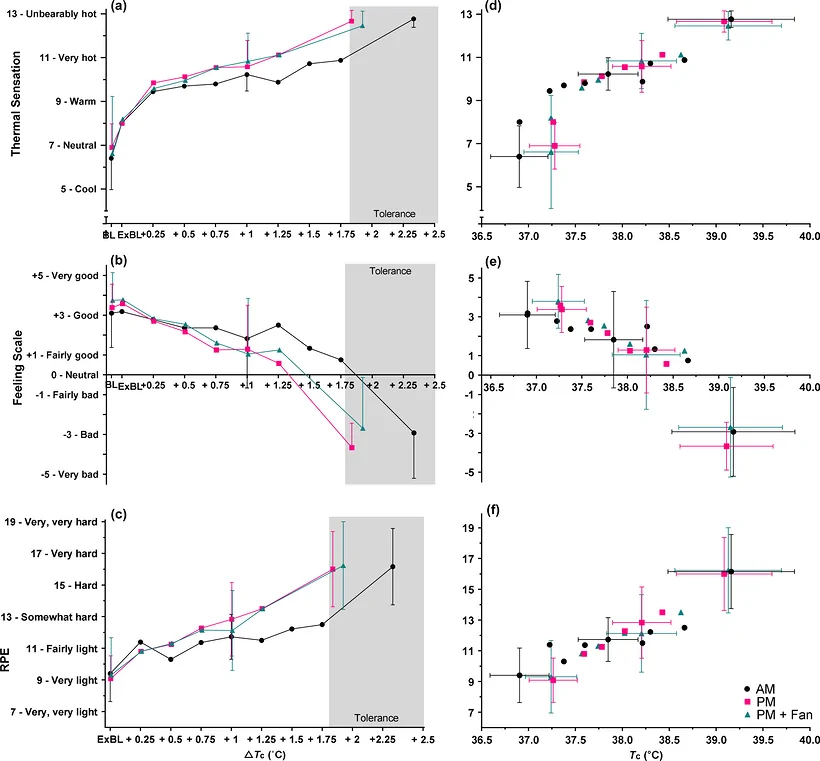
Other relevant perceptions as a function of change in core temperature (Δ *T*_c_; a–c) or the core temperature (*T*_c_; d–f) from exercise baseline (ExBL). *n* = 12 for AM, *n* = 13 for PM and PM+Fan. Data are the mean and SD for *n* = 12 for AM, *n* = 13 for PM and PM+Fan at Resting Baseline, Exercise Baseline and Tolerance, and while *n* ≥ 10 during heat stress. Abbreviations: RPE, Rating of Perceived Exertion; *T*_c_, core temperature.

Thermal discomfort (Figure 7) increased linearly with *T*_c_, at a rate (slopes; Figure 8a) that was not significantly different between conditions (AM, 4.1 ± 3.3 a.u./°C; PM, 4.5 ± 1.9 a.u./°C, *P* = 0.776, *g *= 0.079; PM+Fan, 3.9 ± 1.2 a.u./°C, *P* = 0.315, *g *= 0.362). One participant showed a conspicuously high thermal discomfort sensitivity in AM (i.e., 7 SD above the mean; Figure 8A), which was also their first experimental trial. Omitting that datum would indicate the Discomfort Sensitivity to be lower in AM than PM; 3.3 ± 1.5 vs 4.4 ± 1.9 a.u./°C, p = 0.003, *g* = 1.074. The predicted *T*_c_ threshold for the onset of discomfort was significantly higher (+0.23°C) in PM compared with AM (*P* < 0.001, *g *= 0.407) and was not different when compared with PM+Fan (*P* = 0.860, *g *= 0.119; Figure 8b). The modelled onset of discomfort was approximately baseline *T*_c_ in all three conditions (Figure 8c). Thermal sensation, feeling and RPE also responded linearly with increasing *T*_c_ (Figure 9) at rates that did not differ between conditions (AM vs. PM *P* > 0.194, PM vs. PM+Fan *P* > 0.158).

## 4 DISCUSSION

The purpose of this study was to determine: (1) how naturally occurring differences in baseline *T*_c_ (time of day) affect peak heat tolerance during low‐intensity exercise/work; and (2) what role (if any) opportunity for behavioural thermoregulation had on the physiological mechanisms involved. In support of hypothesis 1, although baseline *T*_c_ was significantly higher in PM compared with AM, this did not confer a higher peak tolerable *T*_c_, and thus the thermal reserve was smaller in the PM. Additionally, the *T*_c_ thresholds for hyperventilation were correspondingly higher in PM, but the sensitivities (slopes) of hyperventilation, cerebral perfusion and thermal discomfort to increasing *T*_c_ did not differ reliably with time of day despite higher peak ventilation in PM. The modelled/predicted thresholds for hyperventilation and thermal discomfort approximated baseline *T*_c_ in both AM and PM. Counter to hypothesis 2, providing an opportunity for behavioural thermoregulation did not reliably alleviate thermal discomfort and thus had no measurable effect on peak thermal tolerance or ventilatory responses, on average, as tested in the afternoon.

### 4.1 Diurnal effects (i.e., effect of a different regulated starting *T*_c_)

Although the effect of an artificially altered baseline thermal state on heat tolerance has been examined in several studies of animal models and humans (e.g., González‐Alonso et al., 1999; Walters et al., 2000), the purpose of the present study was to investigate the effect of regulated fluctuations in *T*_c_ on peak tolerable *T*_c_. Resting *T*_c_ in AM was significantly lower (0.35°C) than in PM (Figure 3), demonstrating a circadian effect of comparable magnitude to previous studies (i.e., 0.3°C–0.5°C) (Atkinson & Reilly, 1995; Hobson et al., 2009; Otani et al., 2020). Yet, the peak temperatures reached were similar (∼39.1°C), thus, the end *T*_c_ rather than its change dictated heat tolerance capacity, at least in this setting of having hot, wet skin and a modest work rate. These findings are consistent with those of Hobson et al. (2009), who also showed a large diurnal increase in starting *T*_c_ in the PM (0.5°C higher), whilst end *T*_c_ remained the same (38.7°C ± 0.9°C) when cycling to exhaustion in the heat. Likewise, Otani et al. (2020) found *T*_c_ to be lower in AM than PM at the outset but not at exhaustion when cycling in the heat (AM, 38.3°C ± 0.4°C; PM 38.6°C ± 0.5°C; *P* = 0.189; *d *= 0.61). Those studies focused primarily on exercise capacity/performance and accordingly used much higher work rates (65% and 70% ${\dot{V}}_{O_{2}\mathrm{peak}}$, respectively) and attained lower final *T*_c_ (by ≥0.4°C) than in the present study. Therefore, it is unlikely that their participants reached heat tolerance per se but rather exercise‐ and heat‐related exhaustion wherein metabolic, cardiovascular and neuroendocrine strain would all have been substantially higher and contributed to an inability to maintain the workload. Although Reilly & Garrett (1998) instead found *T*_c_ to be lower at both the outset and completion of high‐intensity cycling (70% ${\dot{V}}_{O_{2}\mathrm{peak}}$) in AM than in PM, their context was less overtly heat stressful, i.e., limited to 1 h and in a temperate environment. Thus, overall, it appears that a typically higher regulated *T*_c_ in the afternoon does not permit a higher peak tolerable *T*_c_ during uncompensable heat stress with high *T*_sk_, hence heating and work capacity are diminished, as occurs in settings whereby initial *T*_c_ is manipulated (i.e., non‐homeostatic) by precooling or preheating.

Ventilation has been shown to respond linearly to increasing *T*_c_ when exercising in the heat (Fujii et al., 2008; Hayashi et al., 2006; Tsuji, Honda et al., 2016). That linearity was found also in the present study. Two points are notable. First, although linearity was apparent for most individuals [i.e., *r* was >0.90 for the change in ventilation (Δ$\dot{V}$_E_)/Δ*T*_c_ in 29 of 35 trials], a non‐linear response was apparent in some trials and reflected an exaggerated hyperventilation (Figure 5; see also below). Second, ventilation averaged 16% higher at the same end *T*_c_ in PM than in AM (Figure 4; Table 1). Despite the statistically higher ventilation, this had no substantive or reliable effect on $P_{\mathrm{ET},CO_{2}}$ at peak tolerance (Figure 4; Table 1). More importantly, the sensitivity of ventilation and $P_{\mathrm{ET},CO_{2}}$ to *T*_c_ tended to be more severe in PM (Figures 5 and 6). Given that perceptual and other physical indicators of exertion (i.e., heart rate, feeling state, thermal discomfort and RPE) remained the same between AM and PM and that work rates and peak ventilations were modest, it seems unlikely that this increase in ventilation was a result of metabolic factors, feedforward motor command or greater perceptual fatigue. Speculatively, it might have been associated with higher central chemosensitivity to CO_2_.

The *T*_c_ onset threshold for hyperventilation shifted up comparably to the higher resting/regulated *T*_c_, resulting in a tendency to have more severe respiratory responses in the afternoon owing to less thermal reserve. Tsuji, Hayashi et al. (2016) also investigated the slopes and thresholds of respiratory measures (i.e., $\dot{V}$_E_, frequency, tidal volume and $P_{\mathrm{ET},CO_{2}}$) during cycling (50% ${\dot{V}}_{O_{2}\mathrm{peak}}$) in the morning (06:00 h) and the early evening (18:00 h). Likewise, they found that the threshold for hyperventilation (demonstrated via $P_{\mathrm{ET},CO_{2}}$) was higher in the evening. To identify these thresholds, they manipulated starting *T*_c_ using 25 (morning) or 50 min (evening) of cold‐water immersion (18°C) such that *T*_c_ was reduced by 1°C with the onset of exercise. The $P_{\mathrm{ET},CO_{2}}$ thresholds in both the study by Tsuji, Honda et al. (2016) and the present study (36.95°C ± 0.44°C; Figure 6) occurred at or below the typical resting *T*_c_ for that time of day (i.e., between 36.6°C and 37.2°C). It is therefore tempting to suggest that even minor rises in *T*_c_ might stimulate hyperventilation during physical exertion and might do so regardless of the prevailing *T*_c_ with the time of day or intensity of exertion, with a caveat that the thermal stimuli were atypical in the study by Tsuji, Honda et al. (2016) by virtue of the strong precooling and in the present study by virtue of the strong skin heating. Yet, further evidence in support of the notion that hyperventilation develops with the onset of *T*_c_ heating is that similar heating studies have previously shown carotid body activity and sensitivity to be the cause of heat‐induced hyperventilation at rest and in exercise (Gibbons et al., 2022).

Insufficient or excessive CBF could play a role in limiting heat tolerance by virtue of cerebral hyperthermia and cerebral hypoxia (hypoperfusion) or excessive ICP (from relative hyperperfusion) (Bain et al., 2013; Gibbons et al., 2021; Nybo et al., 2002; Oshorov et al., 2021; Sharma, 2006). In the present study, neither CBF nor nICP was altered substantively or systematically at peak heat tolerance (Table 2), but it is also difficult to know what this indicates for them being potential mediators, for several reasons. Findings are mixed on whether CBF is reduced (e.g., Nybo et al., 2002; Trangmar et al., 2014) or unchanged or increased (e.g., Cal, dwell et al., 2020; Gibbons et al., 2021) during exercise under severe heat stress. Although some disparity might reflect regional differences (Bain et al., 2013) or artefact whereby CBF has most commonly been indexed from blood flow velocity in the middle cerebral artery, some disparity is likely to be real and reflect a maintained or increased CBF in studies with lower concomitant stress caused by exercise intensity, orthostatic stress, dehydration (Trangmar et al., 2014) or severity of *T*_c_ heating owing to constraints on ethical end points or duration of exposure. Regardless, it also remains unclear to what extent any reduced CBF is mediated by hyperventilation (Bain et al., 2013; Tsuji et al., 2018; Gibbons et al., 2021) or has much influence on ICP (Cardim et al., 2016; Gibbons et al., 2021). The impact of CBF on brain temperature, a key mediator of heat intolerance (Walters et al., 2000), might also be atypical relative to evolutionary contexts of heat stress by virtue of the artificially high *T*_sk_ incurred within these studies. Specifically, water‐perfused suits, clothing and moderate‐intensity cycling within low airflow and hot laboratory environments promote atypically high *T*_sk_ and thereby elevated arterial temperatures, which would reduce perfusion‐mediated heat extraction from the brain and increase its own heat production (Bain et al., 2013).

Finally, although the ICP response to exercise and exogenous heat stress in healthy populations is largely unknown, the lack of an increase in nICP within the present study (Table 2) is not necessarily at odds with the findings of an elevated nICP at peak heat tolerance in the only study of which we are aware (Gibbons et al., 2021). Gibbons et al. (2021) found nICP at peak tolerance to be elevated relative to a temperate resting state, whereas the baseline measure in the present study was during the exercising baseline. Exercise per se does not increase ICP in people suffering from idiopathic intracranial hypertension (Yiangou et al., 2024), but effects in healthy population appear to be unknown.

### 4.2 The effect of thermal behaviour

Behavioural thermoregulation remains under‐studied as a possible mediator of physiological or tolerance effects of heat stress, but those who have investigated it tend collectively to show that behavioural thermoregulation [whether it be with a fan in an office (Nishihara et al., 2022), a cold‐water‐perfused jacket during exercise (Vargas et al., 2019) or manipulated work rate during exercise (Schlader et al., 2009; Schlader, Simmons et al., 2011)] tends to ease thermal discomfort. However, in the present study, thermal discomfort was not alleviated with behavioural use of a misting fan directed onto the face of participants, nor was the predicted *T*_c_ onset of discomfort or severity of the response altered (Figures 7 and 8); neither were other perceptual measures attenuated with access to the fan (Figure 9). The lack of an effect on discomfort might have occurred because of the overwhelmingly high temperature and wettedness of the much larger non‐facial skin as *T*_c_ continued to rise; i.e., the high *T*_sk_ and combined high skin wettedness nullified any effect of a cooler face. The addition of behavioural cooling also did not increase peak heat tolerance, because final *T*_c_ was not different between the PM (39.11°C) and PM+Fan (39.17°C; *P* = 0.764). The lack of attenuation of thermal discomfort with facial fanning is, however, consistent with the findings of Wang et al. (2023) and perhaps indicates that supplemental or alternative approaches, such as perceptual cooling using menthol (Schlader, Simmons et al., 2011), would have been prudent. Given that there was no evidence of reduced thermal discomfort, the effect of behavioural cooling on heat intolerance remains untested in this study. However, the perception of having behavioural control (i.e., the control over pressing the button) might have influenced the remaining results (described below) more so than the reported thermal discomfort.

With availability of the fan, ventilation at peak tolerance was 14% (6.9 L/min) lower (*P* = 0.046), mediated by lower tidal volume, and sufficient to lessen hypocapnia by an average of 3.4 mm Hg. These findings are in line with previous research showing that ventilation is reduced with the onset of facial cooling (Lucas et al., 2010, 2015). Although ventilatory responses showed far more variability between participants than between PM and PM+Fan, the hyperventilatory response appears to have been blunted for at least two participants (Figure 6), who reached higher *T*_c_ in the fan condition. It remains possible that in some individuals, thermal tolerance and heat‐induced hyperventilation might be driven in part by perceived discomfort, but these findings are observational, and this response was not typical in the present cohort.

### 4.3 Perspectives, limitations and delimitations

The present findings strengthen support for the concept of an individual critical tolerable core temperature during uncompensable heat stress and demonstrate that naturally occurring diurnal differences in starting core temperature alter thermal reserve rather than the maximal tolerable temperature. Practical implications could include the work capacity‐constraining impact of afternoon exposures, exercise or passive warm‐up, or lack of thermal recovery from prior activity. It was also apparent that diurnal effects on heating capacity are far smaller and therefore less relevant than inherent differences between individuals, even within the relatively homogeneous cohort studied here.

The uncompensable heat stress of cycling at a modest work rate was necessary to elicit and thus identify a terminal *T*_c_ and to incur the heat discomfort that will have existed in many published heat stress studies. However, it carried notable limitations that affect generalisability for ecological validity. It creates hot, wet skin, which creates its own cardiovascular, thermoregulatory and psychophysical strain, while also causing the above‐mentioned hotter inflow of blood to the brain than would occur in most real‐life settings (except for spa or sauna bathing, heavy protective clothing, etc.). This feature of the method also means that activation of heat loss effectors, which would respond rapidly, have negligible benefit (for sweating) or are counterproductive (for vasodilatation) for offloading heat and thus extending the thermoregulatory capacity. Therefore, the present atypical heat stress context negates some key benefits of high aerobic fitness and heat acclimation/acclimatisation for heat stress capacity.

Further research might consider the interaction between diurnal effects on thermal reserve and other potential modifiers of heat tolerance, such as personal factors (e.g., sex, menstrual cycle phase, heat acclimatization status, aerobic fitness) and situational factors (e.g., circadian misalignment from shift work and more behaviourally realistic opportunity to regulate the heat stress endogenously or exogenously). Understanding these possible interactions might have implications for occupational and athletic scheduling in hot environments. Extending to more ecologically valid settings [such as prolonged occupational heat exposure (firefighters, bakery and factory workers, construction teams, miners), self‐paced exercise or real‐world heat exposure] will help to determine whether critical core temperature and diurnal modulation of thermal reserve translate to these settings. Such research would also inform evidence‐based recommendations for safer work–rest cycles, appropriate cooling strategies to real work heat exposure, and mitigate the risk of thermal distress across different times of day.

The rectal rather than oesophageal index of core temperature might be considered as a limitation but was used here for multiple reasons: (1) the rectum is valid in severe heat stress because it is a primary source of endotoxins that precipitate heat stroke; (2) the high *T*_sk_, low evaporative capacity and modest rate of heating meant that it would have tracked (Gagnon et al., 2010) and approximated oesophageal temperature (Gibbons et al., 2021, 2022); (3) it is less susceptible to artefacts caused by swallowing water during rehydration or saliva during misted fanning of the face; and (4) it aided in recruitment of participants and minimised a confounding form of psychophysical stress during heating.

The individual variability in ventilatory and hypocapnic responses, particularly in response to facial cooling (Figure 6; PM vs. PM+Fan), highlights the need to understand responder characteristics better. Notable limitations of this study in that context were in not having determined the reliability of either peak heat tolerance or of facial fanning on hyperventilation, and the fanning increased the duration required to attain peak *T*_c_. Another limitation was not assessing the peripheral or central chemosensitivities of participants to help delineate physiological from psychophysical components of hyperventilation. That information would have provided a stronger assessment of hypothesis 2 and, in practical contexts, would help to identify who might benefit vs. potentially be at greater risk from behavioural or sensory cooling strategies under heat stress. Such work might help to address some under‐studied mechanisms of heat intolerance, including individual differences.

## 5 CONCLUSION

The peak tolerable *T*_c_ was not higher in PM than AM, hence the thermal reserve was smaller. Additionally, the thresholds for hyperventilation and thermal discomfort remained at approximately baseline *T*_c_ across the day, although ventilatory sensitivities were relatively high (∼10 L/min/°C) in this setting. Cerebral perfusion was not reliably reduced from baseline even at peak tolerance, despite its primary dependence on the arterial partial pressure of CO_2_, and neither did nICP show any reliable change from its exercising baseline. Stability of CBF and nICP both within and between conditions does not reveal whether they are or are not likely to be mediators of heat intolerance. Opportunity for behavioural thermoregulation did not alleviate thermal discomfort, thus its effect on heat intolerance remains untested/unresolved within this study. Although facial cooling modestly reduced ventilation and attenuated hypocapnia, this did not affect CBF measurably or impact peak heat tolerance. The respiratory responses indicate that, for most individuals, *T*_c_ at peak heat tolerance is unlikely to be dictated by increased autonomy for behavioural thermoregulation.

## AUTHOR CONTRIBUTIONS

James D. Cotter, Travis D. Gibbons and Kate N. Thomas conceived the question and designed the study with Tiarna Stothers. All six authors collected the data at the School of Physical Education, Sport and Exercise Sciences, University of Otago, and contributed to data analysis and interpretation. James D. Cotter and Tiarna Stothers wrote the first draft of the manuscript, and Holly A. Campbell produced the figures. All authors provided critical feedback and approved the final version of this manuscript and agree to be accountable for all aspects of the work in ensuring that questions related to the accuracy or integrity of any part of the work are appropriately investigated and resolved. All persons designated as authors qualify for authorship, and all those who qualify for authorship are listed.

## CONFLICT OF INTEREST

None declared.

## GENERATIVE AI STATEMENT

Generative artificial intelligence was not used in any part of this research or in the manuscript preparation or revision.

## Data Availability

The data will be made available on reasonable request to the corresponding author.
